# Ctrl alt share

**DOI:** 10.1038/sdata.2015.4

**Published:** 2015-02-17

Code and software are now an integral part of research data generation, usage and analysis. Correspondingly, there is an increasing awareness of the need to share code to facilitate reproducibility and validation of published work. *Nature* and the Nature journals recently announced an updated policy with regard to sharing code^1^, and we are pleased to announce today a new code sharing policy for *Scientific Data*, which brings us in line with the Nature journals.

The core of the Nature journal policy is a requirement that authors declare the location and accessibility of all custom code and software that is central in supporting the findings reported in an article. *Scientific Data* will now ask for code availability statements for any custom code used in the generation or processing of datasets described in our articles. This new policy also makes clear that *Scientific Data’s* referees may request access to custom computer code, and our Editorial Board members may require that code be made available when they deem it necessary. Our full policy on computer code availability can found within our editorial and publishing policies (http://go.nature.com/RoEfwu).

To encourage authors to comply with this new policy, we have added a new code availability subsection to the methods portion of our article template (http://go.nature.com/utFVFo). This dedicated code sharing section will make custom code more visible to our referees, and facilitate discovery of the code by readers of the article after publication. Within this section, we also encourage authors to include information on software versions, if relevant, and any specific variables or parameters used to generate, test, or process the current dataset. We believe that this new methods subsection will improve the reproducibility of computational steps within our works and will also assist readers of *Scientific Data* papers in reusing datasets.

Where code and software has been developed specifically for the current study, we consider it best practice to share source code at a level that allows others to fully reproduce the data generation or processing steps. We recommend that authors consider using a version controlled repository (for example GitHub, https://github.com/) and archive a copy of the code used in the generation of the dataset in a DOI minting repository under an appropriate open source licence. This benefits the scientific community by giving researchers the flexibility to build upon source code within the control version repository instance, while maintaining a stable and open version of the code that can be cited through a persistent DOI. Facilitating this process, Zenodo (http://zenodo.org) and figshare (http://figshare.com), two DOI-issuing research repositories, currently provide systems that allow code at GitHub to be directly ported to and archived at their repositories.

Many *Scientific Data* authors already include relevant code in their published work (for example, refs 2–5) and this policy update reflects and supports the current publication practices in many communities. However, given the diversity of practices in the wide range of disciplines we cover, we cannot insist that code and software be shared openly in all cases—although this is preferred. Ultimately, our Editorial Board members and referees will play a central role in helping us enforce this policy in a manner consistent with community expectations. We will continue to work with individual communities to put together best-practice code and software guidelines, to promote the publication of reproducible research.
